# 

**Published:** 1897-08-07

**Authors:** 


					*"? Jim u t>,3i 11 rxj. ? AB&ulu TET7Y?fUKE,?tnereiore?cc.ai. ^uuusi im, 10*t. ^
CADBURY'S -a TV CADBURY'S
COCOA m ipf 1?^' COCOA
" The standard of highest purity at pressnt /-*1? 1 no ALKALIES USED,
attainable.'*? Lancet.
WHICH HOSPl.TAJ.
No. 567. Vol. XXII. [JfgSSlS] Saturday,
August 7th, 1897.
L bSp 316. J PRICE TWOPENCE.

				

